# Supplementary material for: Blocking muscle wasting via deletion of the muscle-specific E3 ligase MuRF1 impedes pancreatic tumor growth
Source: Commun Biol. 2023 May 13;6:519. doi: 10.1038/s42003-023-04902-2 (PMC10183033; doi:10.1038/s42003-023-04902-2)
Supplement: Supplementary file 3 — Description of Additional Supplementary Files [file 42003_2023_4902_MOESM3_ESM.pdf]

Description of Additional Supplementary Files

File name: Supplementary Data 1

Description: All ubiquitination sites annotating to unique proteins retained for follow-up analyses.

File name: Supplementary Data 2

Description: Ubiquitinated sites changed in WT in response to tumor burden.

File name: Supplementary Data 3

Description: 10plex TMT follow-up IPA analyses.

File name: Supplementary Data 4

Description: The overlap between relative protein expression and protein ubiquitination for proteins showing increased ubiquitination in response to tumor burden.

File name: Supplementary Data 5-10

Description: Raw data and follow-up statistical analyses for tumor, serum and muscle metabolomics.

File name: Supplementary Data 11

Description: The data used to generate figures
